# Next Step in Cost Containment of Public Hospital Economy Could Be Merging of Anesthesia and Surgery Budgets

**DOI:** 10.3389/fsurg.2016.00041

**Published:** 2016-07-19

**Authors:** Jacob Rosenberg, Thomas Fuchs-Buder

**Affiliations:** ^1^Department of Surgery, Herlev Hospital, University of Copenhagen, Copenhagen, Denmark; ^2^Department of Anaesthesia, CHU de Nancy, Hôpitaux de Brabois, Nancy, France

**Keywords:** OR management, cost control, public hospital, budgets, anesthesia, surgery department, hospital

Public hospitals are typically run by diagnosis-related groups (DRG) as income. There is, however, a major problem in that medical reimbursement per patient is dropping and at the same time the surgery-related costs have increased remarkably in recent years. It is therefore necessary to optimize income as well as reduce expenses as much as possible.

The major part of the expenses for a patient undergoing, e.g., laparoscopic rectal resection is divided by the intervention stage covering approximately 50% of the total expenses and the ward stay covering approximately 35% of the total expenses (1). It is therefore natural to focus on lowering ward stay as well as looking at cost containment during surgery and anesthesia. There are ongoing efforts to reduce ward stay in for instance optimized care regimens, so called fast-track surgery, and recently in the setting of the Perioperative Surgical Home that employs close collaboration between anesthesiology, surgery and nursing in order to send the patient home as early as possible in good shape.

Only a very small amount is spent on anesthesia drugs, whereas about 20 times more are spent on surgical devices (1). Thus, the total intervention stage expense for a laparoscopic rectal resection was 5,491 Euro where anesthesia only cost 123 Euro, and surgical devices cost 2,361 Euro (1). It is therefore important to negotiate better prices for surgical single use equipment, whereas a slight increase in anesthesia expense would not make a big difference to the overall budget. Thus, if anesthesia expenses would be increased in order to get the patients out of the operating room faster, then it would not matter much for the overall calculations and it would in the end be possible to run an extra case in that operating room. An example of this is the use of sugammadex for reversal of muscle relaxation in general anesthesia where the patient will be out of relaxation and anesthesia extremely fast, and thereby makes it possible to perform an extra surgical case if properly managed. Thus, a study (2) and a recent health economic assessment (3) have shown that there may be an overall economic benefit, even when spending more money on general anesthesia. In line with this, it is evident that inefficient scheduling of operating room time resulting in delays of surgery and even in cancelations will be very costly for the hospital (4) and especially turn over time between cases has a major impact of the overall operating room efficiency.

A major problem in typical operating room management is that because of working schedule, it is not possible to start a surgical case in the afternoon after a certain time point. The reason is fear of running overtime. However, it is evident that such a scenario would actually increase the surgical per case cost (5). The reason for this is that such a policy for operating room management will result in multiple cancelations of surgical cases with a subsequent reduced income to the hospital. In the current economic model, in most public hospitals run by DRG income the surgical department will receive 100% of the DRG income per case, and the anesthesia department is funded with a fixed budget decided by the hospital management. Thus, surgeons are bringing the income, whereas the anesthesiology department will be punished if they run overtime. There is therefore no incitement for the anesthesia department to push that extra afternoon case through, because it will bear a risk of running overtime and paying more money for salaries. There is therefore an important conflict of interest between the surgery and the anesthesia department in the daily running of the OR environment, simply because the budgets are separated. Ideally, and in some countries, DRG “weights” and thus reimbursement is determined by historical costs from, e.g., the past 3 years calculated by, e.g., the cost per patient (CPP) method. This makes the reimbursement more appropriate although technical improvements may not be covered. Another similar model with the same goal is that the surgical department buys service from the anesthesiology department including active operating minutes.

In other situations, it makes perfectly sense to increase collaboration between anesthesia and surgery (6), and for instance when developing principles of the Perioperative Surgical Home and other aspects of perioperative care, it gives perfectly good sense to work for a very close collaboration between the two specialties. Moreover, from a perioperative perspective of patient care, the separation on an anesthesiological and surgical contribution to patients’ outcome becomes increasingly artificial. The implementation of the ERAS-protocol may lead to up to 50% reduction of postoperative complications (7). This protocol contains both surgical specific components such as “no drains” and anesthesia specific components such as “mid thoracic epidural analgesia.” The implementation of such perioperative approaches could be facilitated if financing comes from a common perioperative budget. Another example is the prevention of adhesions after laparoscopic surgery where lower intra-abdominal pressure may be beneficial (8) and one strategy to maintain surgical working space despite lower insufflation pressure is deepening neuromuscular block (9). Thus, the prevention of surgical complications may increase anesthesia-related costs and a common budget would facilitate such an integrated perioperative approach.

In conclusion, most hospitals are facing economic challenges in that income based on diagnosis-related groups are decreasing and expenses are increasing. We therefore have to optimize both income and expenses. This can be obtained by a closer collaboration between surgery and anesthesia, and optimally by a full integration of budgets for the two departments. In this model, it will be favorable for all stakeholders to work for very short turn over time between surgical cases and utilizing the operating room to its absolute maximum by operating to the end of the day, and not stopping in the early afternoon in fear of running overtime. Expenses for general anesthesia are microscopic compared with expenses for the surgical procedure, and it may therefore be acceptable to increase cost of anesthesia provided that it would result in a faster turn over giving room for extra cases.

## Author Contributions

Both authors conceived the idea for the manuscript, wrote the draft, made critical revisions, and approved the final manuscript for submission.

## Conflict of Interest Statement

Dr. JR reports grants from Johnson & Johnson, grants and personal fees from Bard, personal fees from Merck, outside the submitted work; Dr. TF-B declares personal fees from Merck, outside the submitted work.
